# Genetic and molecular characterization of a novel reassortant H3N2 influenza virus from a sick pig in Eastern China in 2019

**DOI:** 10.1186/s13567-025-01462-7

**Published:** 2025-02-10

**Authors:** Fan Yang, Linfang Cheng, Fumin Liu, Hangping Yao, Nanping Wu, Lihua Xu, Haibo Wu

**Affiliations:** 1 State Key Laboratory for Diagnosis and Treatment of Infectious Diseases, and National Clinical Research Center for Infectious Diseases, School of Medicine, the First Affiliated Hospital, Zhejiang University, Hangzhou, 310003 China; 2 Animal Husbandry and Veterinary Institute, Zhejiang Academy of Agricultural Science, Hangzhou, 310021 China

**Keywords:** Swine influenza virus, H3N2, reassortant, Eastern China

## Abstract

Swine influenza viruses (SIVs) cause clinical respiratory symptoms associated with high mortality rates among pigs. Because pigs can be a “mixing vessel” for influenza viruses, the SIV might pose a serious threat to animal and human health. In this study, an H3N2 SIV [A/swine/Zhejiang/19/2019(H3N2) (ZJ-SW19)] was isolated from a sick pig in Eastern China in 2019, and its molecular genetics were characterized. Phylogenetic analysis demonstrated the hemagglutinin (HA) and neuraminidase (NA) segments of ZJ-SW19 are highly homologous with those of H3N2 SIVs, belonging to human-like lineages; in contrast, the remaining six SIV segments (PB2, PB1, PA, NP, M, and NS) demonstrate the highest similarity with H1N1 SIVs isolated in East Asia during 2014–2020. The in vitro analysis of the virus’s growth kinetics revealed that ZJ-SW19 can replicate efficiently in various mammalian and avian cell lines (including MDCK, A549, and DF-1). The receptor-binding analysis results indicated that ZJ-SW19 can bind to human-like receptors (α-2,6-linked sialic acid) and avian-like receptors (α-2,3-linked sialic acid). Moreover, ZJ-SW19 demonstrated significant differences compared with avian- and human-origin H3N2 influenza viruses in the antigenic analysis. Finally, in the pathogenicity test, ZJ-SW19 effectively replicated in the mouse lungs with moderate virulence. Therefore, continuous circulation of novel reassortant H3N2 SIVs indicates the need for long-term, close surveillance of influenza viruses in pig herds.

## Introduction

Influenza A viruses are enveloped viruses containing eight RNA segments, which can infect several host species, including poultry, wild birds, and marine and terrestrial mammals [1]. Swine influenza virus (SIV) is highly contagious, with the ability to infect pigs and cause various respiratory symptoms, such as runny nose, cough, and fever [2]. Although SIV-related clinical signs are typically mild, co-infection of SIVs and other pathogens increases mortality in infected pigs or leads to stillbirths in infected pregnant sows [3, 4]. In pigs, the respiratory tract carries both N-acetylneuraminic acid-α-2,3-galactose (an avian-like receptor) and N-acetylneuraminic acid-α-2,6-galactose (a human-like receptor) [5]. Therefore, pigs are generally considered “mixing vessels” between birds and humans during the evolution, reassortment, and transmission of influenza viruses [6]. Therefore, SIVs circulating in a pig herd can reassort with avian or human influenza viruses to produce novel strains, which can transmit back to humans or pigs, potentially leading to an epidemic or pandemic [7].

Based on multiple antigenic determinants, SIVs isolated from pigs worldwide have been classified into three main subtypes: H1N1, H1N2, and H3N2 [8]. The human-origin H3N2 SIV was first simultaneously identified in pig and human populations in China in 1968 [9]. Subsequently, a reassortant swine-origin H1N1 strain, pdm/2009, first isolated in North America, caused a pandemic in humans; its evolution involved multiple reassortments of avian-, swine- and human-origin viruses [10]. Reassortant SIVs containing segments of pdm/2009 have appeared widely in Southern China, North America, and Eurasia. After the 2009 pandemic, the H1N1 pdm/2009 virus continued to transmit to pigs in many regions and reassort with local viruses, significantly increasing and enriching the diversity of SIV gene sources among pig populations [11]. As such, since the emergence and circulation of the 2009 H1N1 pandemic virus, SIV surveillance has been strengthened globally [12–14]. In 2011, the first known human-origin H3N2 SIV was confirmed in the United States; it caused > 400 human infections over 2011–2018 [15]. In China, H3N2 SIVs were also detected in humans and caused sporadic human infections [16]. Therefore, the widespread transmission of the aforementioned H3N2 SIVs indicates that the virus may be a serious threat to global public health.

In China, the rapid, intensive growth of swine and poultry farming industries may increase influenza virus reassortment frequencies and infection risks [17]. However, available data regarding the genetic and molecular characteristics of H3N2 SIVs in Eastern China are scant; therefore, monitoring influenza among pig populations in Eastern China is crucial. In the current study, we conducted SIV surveillance in Zhejiang, Eastern China; we isolated and characterized the SIV A/swine/Zhejiang/19/2019(H3N2) (ZJ-SW19) to understand the genetic evolution and biological characteristics of SIVs further.

## Materials and methods

### Sample treatment and virus isolation

From December 2018 to January 2019, we performed SIV surveillance in Zhejiang, Eastern China. We collected 125 lung tissue samples from clinically ill pigs and stored them in 1 mL of phosphate-buffered saline (PBS, pH 7.4) containing penicillin G and streptomycin sulfate.

Virus isolation was conducted in embryonated specific-pathogen-free (SPF) eggs, as described elsewhere [18]. In brief, all virus samples were centrifuged; the supernatants were collected, filtered, and injected into 9-day-old SPF eggs, followed by incubation at 37 °C for 3 days. Finally, the allantoic fluid was harvested and evaluated using a hemagglutination inhibition (HI) assay [19]. All positive allantoic fluids were stored and subsequently subjected to genome sequencing.

### RNA extraction and genome sequencing

TRIzol reagent (Life Technology, USA) was used for viral RNA extraction, according to the manufacturer’s instructions, as described previously [20]. In brief, allantoic fluid was mixed with TRIzol reagent for protein lysis. Chloroform was then added to separate the organic and inorganic fractions. After centrifugation, the upper inorganic phase was aspirated and treated with isopropanol for precipitation. The precipitate was washed twice with 75% ethanol and eluted with RNase-free water. Finally, RNA concentrations were detected based on absorbance measured on NanoDrop.

The reverse transcription polymerase chain reaction (RT-PCR) with Uni12 primers, 5′-AGCAAAAGCAGG-3′, was used to synthesize cDNA, as described previously [21]. Eight segments of SIV were amplified separately using specific segment primers [22]. The RT-PCR products were sequenced using the Sanger method. Sequences of each segment of the isolated H3N2 virus were aligned using Basic Local Alignment Search Tool (BLAST), analyzed on BioEdit, and submitted to GISAID (EPI3528504- EPI3528511).

### Phylogenetic study and molecular analysis

Classical reference viruses were selected based on previous reports [8–11, 13, 14]. Moreover reference sequences of the strains used in this study were obtained from the Influenza Sequences Database. Molecular Evolutionary Genetics Analysis was used to construct phylogenetic trees by using the distance-based neighbor-joining method and 1000 bootstrap replications [23].

### Immunofluorescence assay

To evaluate virus activity in MDCK cells, we performed an immunofluorescence assay (IFA), as described previously [24]. We cultured MDCK cells in six-well plates until they formed a confluent monolayer and washed them with PBS twice. Next, we infected the cells with ZJ-SW19 [multiplicity of infection (MOI) = 0.1] for 16 h and washed them twice with PBS. The infected MDCK cells were then fixed with 4% paraformaldehyde and permeabilized using 0.5% Triton X-100. The cells blocked by 3% bovine serum albumin (BSA) were incubated with monoclonal antibodies against NP (GeneTex, USA) at 4 °C overnight. Next, the cells were incubated with Alexa Fluor 488 goat antimouse antibodies (Abcam, USA) at 37 °C for 1 h. The fluorescent signal was detected under an EVOS M7000 Microscope Imaging System (Invitrogen, USA).

### Analysis of virus replication in vitro

We evaluated the growth kinetics of H3N2 SIVs in MDCK, A549, and DF-1 cells [25]. Monolayer confluent MDCK, A549, or DF-1 cells were grown in six-well plates and infected with ZJ-SW19 (MOI = 0.01). The cells were cultured in Dulbecco’s modified Eagle medium containing BSA and tosyl phenylalanyl chloromethyl ketone-treated trypsin. The cultures were centrifuged at 0, 12, 24, 36, 48, 60, and 72 h post-infection (hpi), and their supernatants were collected. Finally, the viral titers were measured using the 50% tissue culture infective dose (TCID_50_) assay [26].

### Receptor-binding analysis

To analyze the receptor-binding specificity of H3N2 SIVs, we performed the HI assay by using receptor-specific red blood cells (RBCs) [27]. In brief, normal chicken RBCs containing both α-2,6 and α-2,3 receptors were prepared, and they were treated with α-2,3-specific neuraminidase (NA) to induce the production of sialidase-treated chicken RBCs, which only contain the α-2,6 receptor. Finally, to define the HA titer, we performed the HI assay by incubating two-fold serially diluted viruses with RBCs in 96-well microtiter plates.

### Antigenic study

The antigenic relationship between ZJ-SW19 and other H3N2 influenza viruses (including those of swine, human, and avian origin) was evaluated using cross-reactive HI assays, as described previously [28]. Mouse antisera for ZJ-SW19, A/swine/Zhejiang/6/2010(H3N2) (ZJ-SW6), A/Hong Kong/2671/2019 (H3N2) (HK-2671), A/Texas/50/2012(H3N2) (Tex-50), A/duck/Zhejiang/4613/2013(H3N2) (ZJ-4613), and A/chicken/Zhejiang/139/2016 (H3N2) (ZJ-139) were generated and stored in our laboratory [22, 29].

### Animal study

To evaluate SIVs’ pathogenicity and replication ability in mammalian hosts, we used 15 6-week-old female BALB/c mice and inoculated them with 50 µL of suspension containing 10^5.0^ or 10^6.0^ 50% egg infectious dose (EID_50_) of ZJ-SW19 via nasal drip [28]. At 3, 6, and 9 days post-infection (dpi), we euthanized three mice, collected their tissues (including lungs, brains, hearts, livers, kidneys, and spleens), and determined host SIV distribution by using the TCID_50_ assay. Moreover, we measured the daily survival rate and weight loss in the remaining six mice over 14 dpi, as reported previously [28]. For histopathological analysis, half of the lung tissue specimens were fixed with formalin solution fixation, embedded in paraffin for 3 days, sectioned, and stained with hematoxylin and eosin (H&E) dye.

## Results

### Virus isolation and molecular analysis results

**Figure 1:**
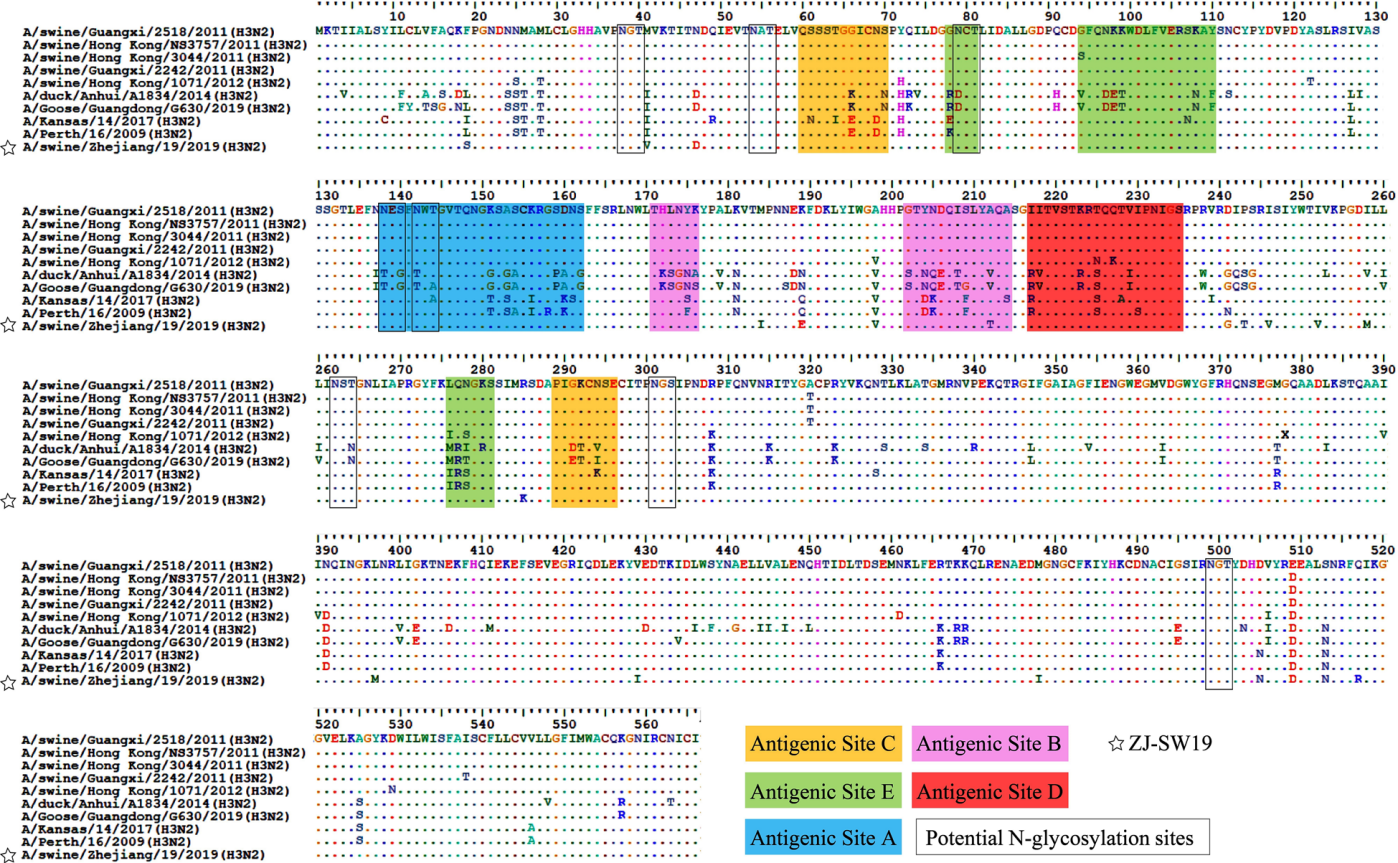
**Amino acid comparison at the HA domains of H3N2 influenza viruses.** Dots denote amino acids similar to the consensus, and squares denote conserved amino acid residues at the receptor-binding site. The asterisk denotes ZJ-SW19. N-glycosylation sites are presented in boxes. Amino acid residues mapped at previously defined antigenic sites A, B, C, D, and E are presented in blue, purple, yellow, pink, and green, respectively.

We first isolated the SIV ZJ-SW19 from grow-finish pigs with clinical respiratory symptoms in Hangzhou, Zhejiang, China, and sequenced its eight segments. Our results indicated that the HA gene is 1701 bp long and encodes 566 amino acid residues (Figure 1). The motif of the HA cleavage site is PEKQTR↓G, indicating that ZJ-SW19 has low pathogenicity [30]. Compared with A/swine/Guangxi/2518/2011(H3N2) (the closest reference), ZJ-SW19 demonstrates 13 amino acid mutations located in HA1 protein (F19S, M41V, N47D, M184I, K184E, A198V, A212T, D241G, P243T, I246V, I252V, I258M, and R285K) and seven in HA2 protein (L397M, V429I, M478I, D505N, E509D, S513N, and Q517R). The amino acid mutation A212T is located in the antigenic site B of HA, which can increase human H3N2 influenza virus replication in eggs [31]. Moreover, the HA protein of ZJ-SW19 contains eight potential N-glycosylation sites: NGT(38–40), NAT(54–56), NCT (79–81), NES(138–140), NWT(142–144), NST(262–264), NGS(301–303), and NGT(499–501). None of these substitutions related to ZJ-SW19’s virulence and host range were observed in PB2 (E627K and D701N) or PA (T97I), and none of the substitutions in NA (H274Y) were associated with oseltamivir resistance [32].

### Phylogenetic analysis results

**Figure 2:**
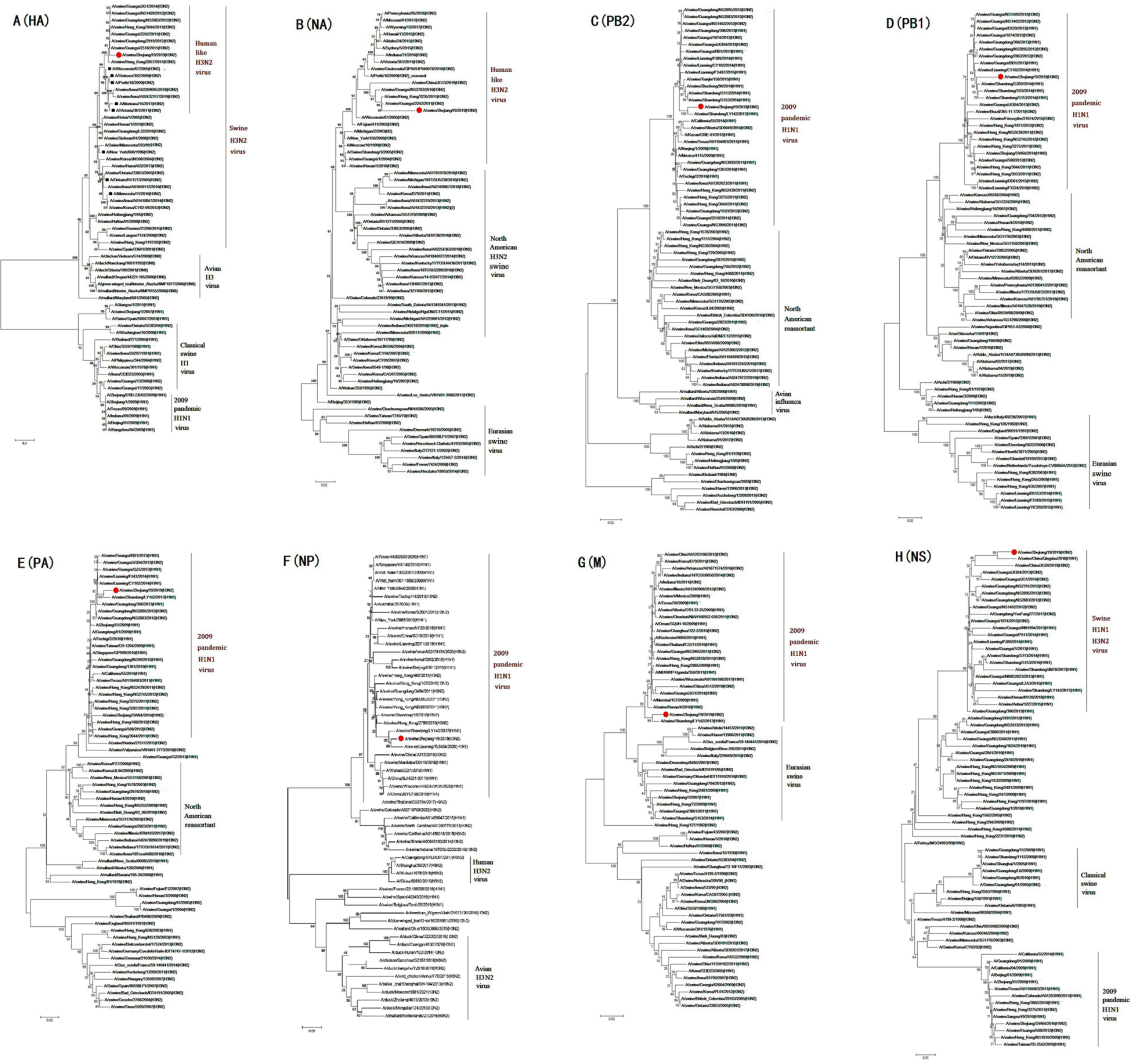
**Phylogenetic trees for the HA(A), NA(B), PB2(C), PB1(D), PA(E), NP(F), M(G), and NS(H) genes in ZJ-SW19 isolated in this study.** Red dots denote ZJ-SW19, and the black box indicates human influenza viruses originating from SIVs. The scale bar represents the distance unit between the sequence pairs.

We determined sequence similarity through BLAST analysis and selected sequences with high homogeneity. The results indicated that the segments of the major capsid proteins (HA and NA) of ZJ-SW19 are closely related to swine H3N2 viruses, whereas the other six segments have the highest homogeneity with H1N1 SIVs isolated in East Asia over 2014–2020 (Table 1). The PB2, PB1, PA, NP, and M segments of ZJ-SW19 were assigned to the H1N1 pdm/2009 lineage, whereas the HA gene of ZJ-SW19 demonstrated the highest homology with A/swine/Guangxi/2518/2011(H3N2). The phylogenetic analysis revealed that the HA segment of ZJ-SW19 has a human-like lineage (Figure 2), and that the NA segment of ZJ-SW19 has the highest homology with A/swine/Shandong/15/2018(H3N2). Taken together, these results demonstrated that ZJ-SW19 is a reassortant strain derived from other local SIVs, particularly H1N1 and H3N2 SIVs; moreover, the internal genes of H1N1 pdm/2009 influence H3N2 SIV prevalence in pig populations.

**Table 1:** **Comparison of ZJ-SW19 genomes with reference virus sequences available in the GenBank database**

Segment	Position	GenBank accession number	Virus with highest nucleotide identity percentage	Homology (%)	Influenza virus lineage
PB2	1–2280	MN416391.1	A/swine/Shandong/LY142/2017(H1N1)	98.38	H1N1 pdm/2009
		MN393805.1	A/swine/Liaoning/PJ89/2014(H1N1)	97.89	
		MN393757.1	A/swine/Liaoning/FS487/2015(H1N1)	97.85	
PB1	1–2274	OL744679.1	A/swine/Anhui/HD21/2020(H1N1)	98.02	H1N1 pdm/2009
		OL311382.1	A/swine/Liaoning/HLD1795/2020(H1N1)	97.93	
		MN393726.1	A/swine/Liaoning/CY102/2014(H1N1)	97.85	
PA	1–2151	MN416578.1	A/swine/Shandong/LY142/2017(H1N1)	98.19	H1N1 pdm/2009
		MK587713.1	A/swine/China/Qingdao/2018(H1N1)	98.19	
		MN393799.1	A/swine/Liaoning/PJ43/2014(H1N1)	98.09	
HA	1–1701	KM028047.1	A/swine/Guangxi/2518/2011(H3N2)	96.41	Human-like H3N2
		KM028807.1	A/swine/Hong Kong/NS3757/2011(H3N2)	96.36	
		KM029423.1	A/swine/Hong Kong/1284/2012(H3N2)	96.24	
NP	1–1497	MN418795.1	A/swine/Shandong/LY142/2017(H1N1)	98.33	H1N1 pdm/2009
		OL311012.1	A/swine/Liaoning/TL5404/2020(H1N1)	98.06	
		KM028664.1	A/swine/Hong Kong/NS3030/2011(H3N2)	97.60	
NA	1–1380	OL439948.1	A/swine/Shandong/15/2018(H3N2)	96.74	Human-like H3N2
		ON850009.1	A/swine/Hong Kong/2789/2013(H3N2)	96.52	
		KM028017.1	A/swine/Guangxi/2242/2011(H3N2)	96.31	
M	1–982	MN418875.1	A/swine/Shandong/LY142/2017(H1N1)	98.46	H1N1 pdm/2009
		OL744684.1	A/swine/Anhui/HD21/2020(H1N1)	98.36	
		CY061768.1	A/swine/Hong Kong/2885/2009(H1N1)	97.60	
NS	1–838	OL311131.1	A/swine/Liaoning/CY1833/2020(H1N1)	98.90	Swine H1N1
		MK587718.1	A/swine/China/Qingdao/2018(H1N1)	98.29	
		OL744685.1	A/swine/Anhui/HD21/2020(H1N1)	97.80

### Growth kinetics

**Figure 3:**
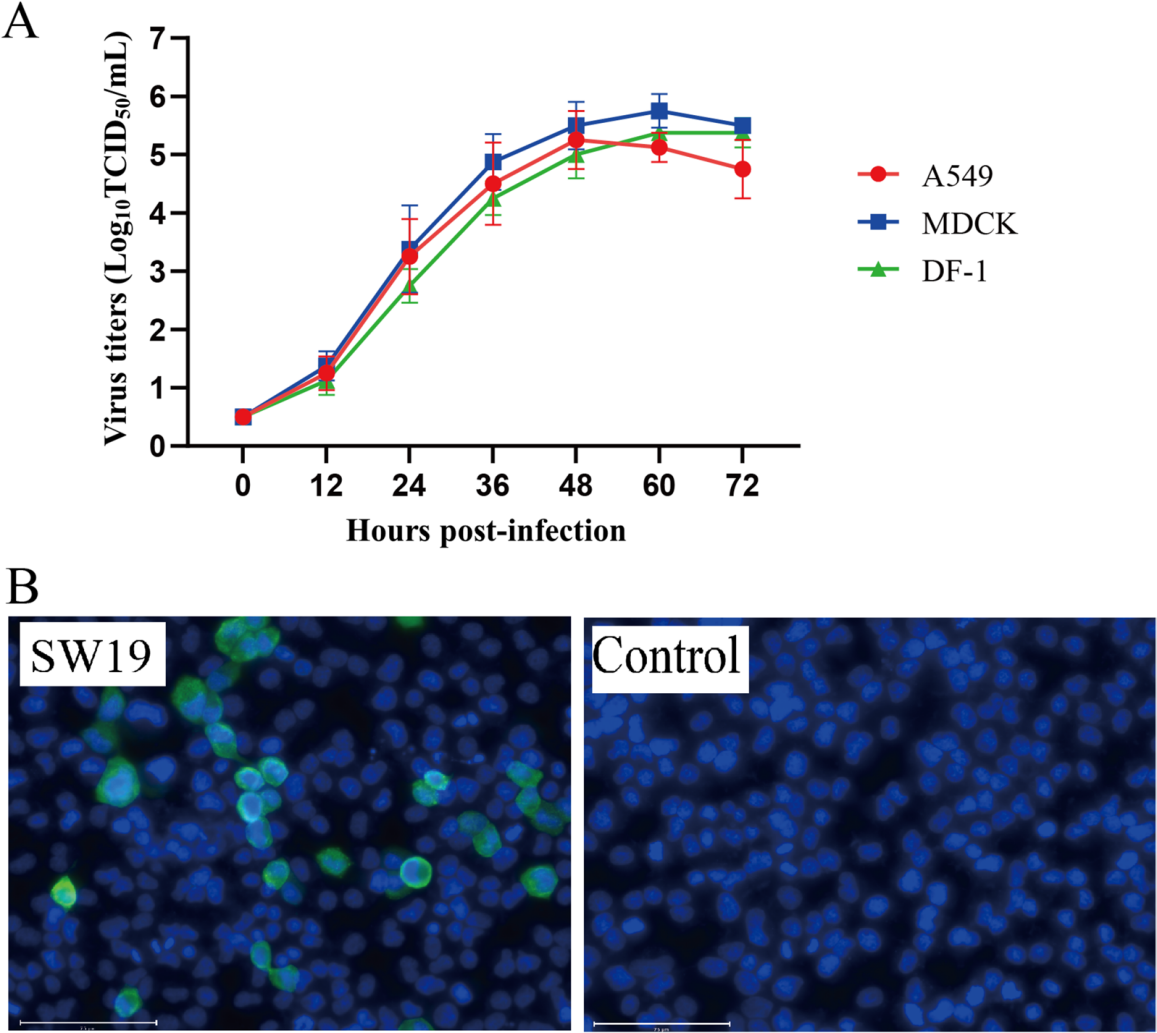
**ZJ-SW19 replication potential in vitro. A** Growth curves of ZJ-SW19 in MDCK, A549, and DF-1 cells (MOI = 0.01). **B** Infection potential of ZJ-SW19 in MDCK cells using IFA.

The replication ability of ZJ-SW19 was evaluated in MDCK, A549, and DF-1 cells. The results of in vitro growth kinetics showed that ZJ-SW19 equivalently replicates efficiently in mammalian and avian cells (Figure 3A). At 12 hpi, live viruses were detected in the cell supernatant. Between 12 and 48 hpi, virus production gradually increased; thereafter, the virus content in cell supernatants remained stable or decreased slightly. Finally, we assessed the infection potential of ZJ-SW19 using an IFA, and the results demonstrated that ZJ-SW19 can effectively replicate in MDCK cells (Figure 3B).

### Receptor-binding analysis outcomes

The receptor-binding specificity assay results suggested that ZJ-SW19 efficiently binds to both human- and avian-like receptors (α-2,6 and α-2,3, respectively). Thus, ZJ-SW19 can infect via either of these two receptor types (Table 2).

**Table 2:** **Virus receptor binding specificity of ZJ-SW19**

Virus	Receptor binding specificity		
	α-2,3	α-2,6 and α-2,3	α-2,6
ZJ-SW19	8	128	128
A/pigeon/Zhejiang/727097/2014(H5N1)	256	256	< 2
A/Puerto Rico/8/1934(H1N1)	< 2	128	128

We treated 1 mL of 10% chicken RBCs with 1000 IU of α-2,3-specific NA at 37 °C for 1 h to produce sialidase-treated chicken RBCs, which only contained α-2,6 receptor. Normal chicken RBCs containing both α-2,6 and α-2,3 receptors were also prepared. To determine specific receptors on treated and untreated RBCs, the avian influenza virus A/pigeon/Zhejiang/727097/2014(H5N1) and human influenza virus A/Puerto Rico/8/1934(H1N1) were included in the receptor binding assay as controls. The HI assay was performed in 96-well plates by incubating 50 µL of two-fold serially diluted viruses with 0.5% RBCs.

### Antigenic study results

Our analysis of the antigenic relationship between ZJ-SW19 and other H3N2 influenza viruses (including those of swine, human, and avian origins) demonstrated that viruses with the same host origin have similar antigenicity, whereas swine-origin H3N2 and human-origin H3N2 viruses have significantly different antigenicity (Table 3).

**Table 3:** **Analysis of ZJ-SW19 antigenic characteristics using HI assay**

Targets of polyclonal antiserum titers	ZJ-SW19	ZJ-SW6	HK-2671	Tex-50	ZJ-4613	ZJ-139
ZJ-SW19	640	320	40	40	< 20	< 20
ZJ-SW6	320	640	40	40	< 20	< 20
HK-2671	40	20	320	160	< 20	< 20
Tex-50	40	40	160	320	< 20	< 20
ZJ-4613	< 20	< 20	40	40	320	320
ZJ-139	< 20	< 20	40	40	320	320

### Pathogenicity in mice

**Figure 4:**
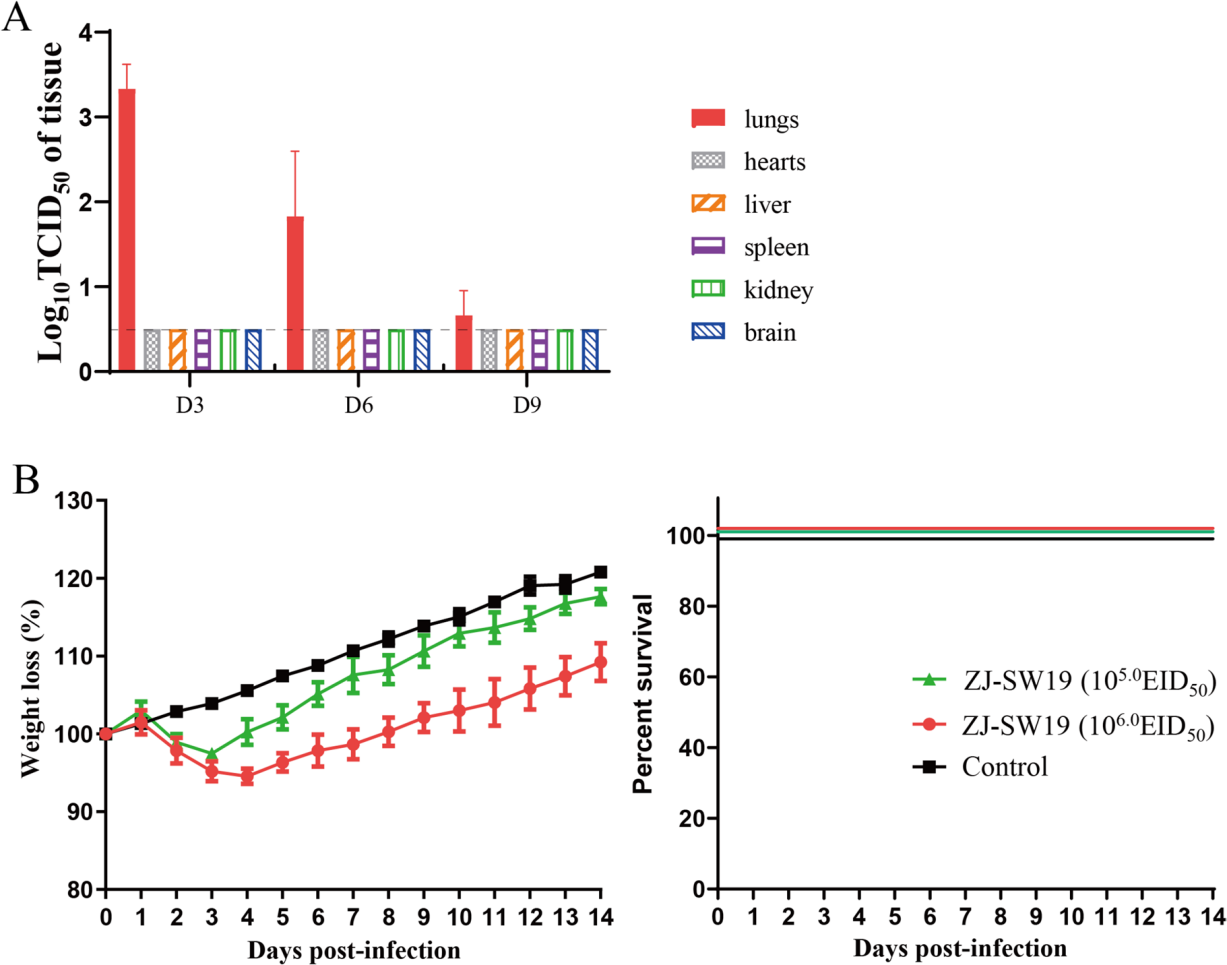
**ZJ-SW19 pathogenicity in vivo. A** Viral titers in organs in the group of 10^6.0^ EID_50_ identified using the TCID_50_ assay. **B** Daily weight loss and survival rate in six mice in each experimental group infected with 50 µL of viruses at 10^5.0^ or 10^6.0^ EID_50_ over 14 dpi.

**Figure 5:**
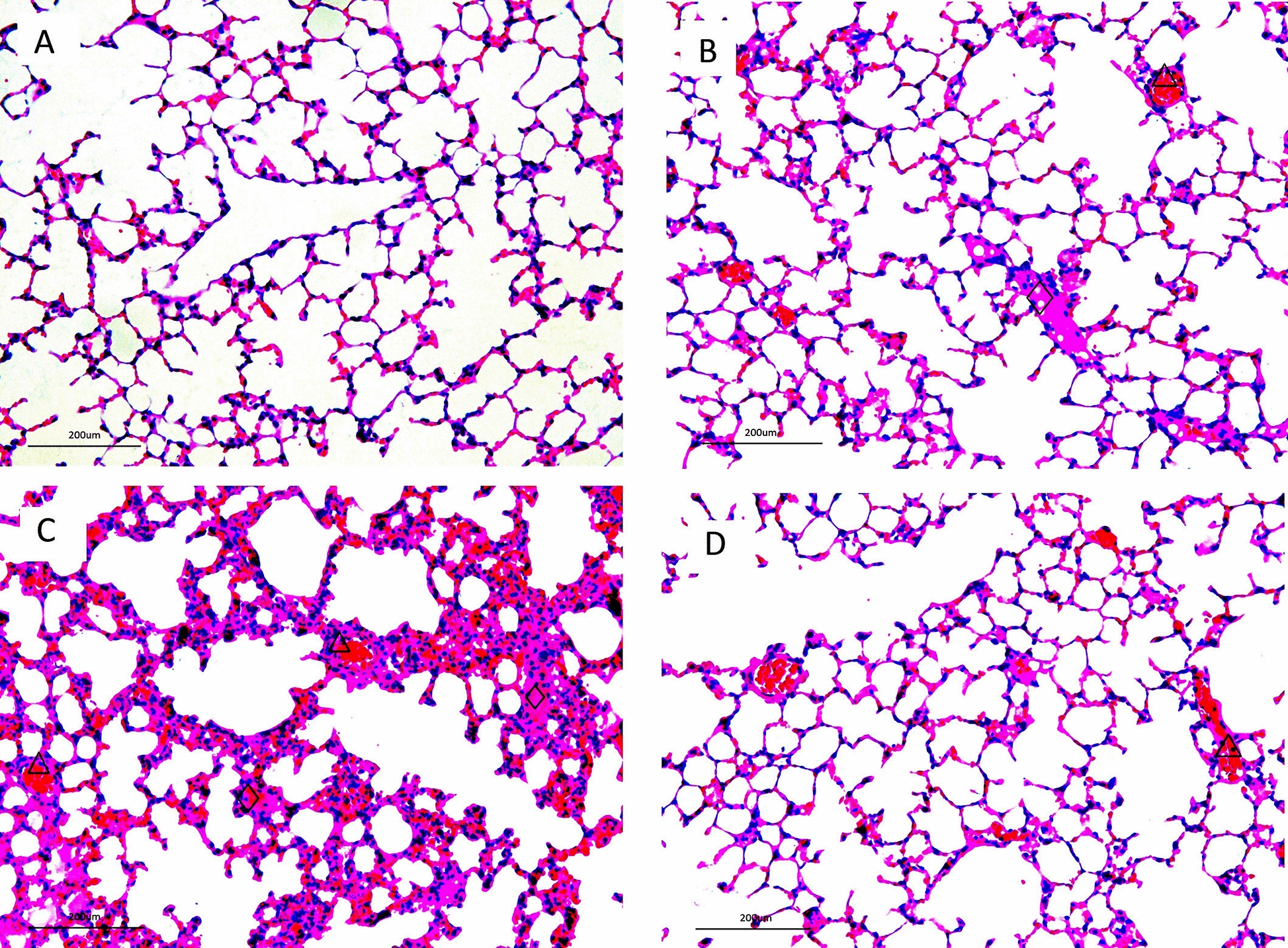
**H&E staining for histopathological analysis of lung tissues of mice infected with 10**^**6.0**^** EID**_**50**_** of ZJ-SW19 on 3, 6, and 9 dpi.** Mice displayed mild or moderate interstitial inflammatory congestion and exudative lesions, accompanied by inflammatory cells (diamond) and RBC infiltration (triangle). **A**, **B**, **C**, and **D** show sections from the control, 3 dpi, 6 dpi, and 9 dpi groups, respectively.

BALB/c mice, the conventional animal model widely used to study influenza virus pathogenesis [20, 26–28], was used to predict SIV pathogenicity and replication potential in mammals. At 3, 6, and 9 dpi, ZJ-SW19 was detected in the lungs of infected mice, with the highest titer being noted on day 3, indicating that ZJ-SW19 can effectively replicate in the lungs without prior adaptation (Figure 4A). In contrast, ZJ-SW19 was not detected in other organs such as the brain, heart, liver, kidneys, and spleen. Moreover, over 5–8 dpi, the infected mice experienced moderate weight loss (4–8%) with 100% survival (Figure 4B). Finally, histopathological analysis revealed multiple plaque lesions in the mouse lung tissue on 6 dpi, indicating mild or moderate interstitial inflammatory congestion and exudative lesions, accompanied by the infiltration of many inflammatory cells and RBCs (Figure 5).

## Discussion

Influenza is the most widely distributed acute zoonotic infectious disease caused by influenza virus. The range of influenza virus hosts is extremely wide, including humans and most animals (e.g., pigs and birds). The H3N2 SIV is one of the most predominant influenza virus strains in pig herds. At large-scale pig farms, swine influenza is a common and difficult-to-eradicate mass disease; it not only causes the death of affected pigs directly but also reduces their production performance, increases their feeding costs, affects the health status and quality of pig herds directly, and poses a great threat to the pig farming industry [33, 34]. H3N2 SIVs are globally endemic, and serological surveillance has revealed that the H3N2 SIV-positivity rate in pig populations is 7.9% [35]. In China, workers coming into contact with pigs have an extremely high risk of epidemic SIV infections. Serological studies have demonstrated that approximately 31.5% of pigs have been infected with at least one SIV type (H1N1 or H3N2 SIV) [36]. In a ferret model, seasonal trivalent inactivated influenza vaccine was noted to be ineffective at preventing H3N2 SIV infection and transmission [37]. Therefore, intensive surveillance is essential for reducing SIV infection and pandemic risk.

In the present study, the H3N2 SIV ZJ-SW19 was isolated, and its antigenicity appeared to differ from that of current human H3N2 viruses. The binding of surface HA protein to the host cell sialic acid can initiate influenza virus infection, and several single amino acid substitutions on HA can affect the host species. The single amino substitution A196T can increase virus replication in eggs without affecting virus antigenicity [31]. The mutations E190D and Q226L in H3 HA and G225D/E in H1 HA are essential for alternations in viral receptor-binding properties during the adaptation of avian viruses to humans and pigs [38, 39]. Here, we noted that ZJ-SW19 binds strongly to the human-like sialic acid receptor but weakly to the avian-like sialic acid receptor. In addition, considering the differences in their biological characteristics, novel reassortant influenza virus strains may also demonstrate considerable differences in pathogenicity and replicability [40]. Finally, we also noted that ZJ-SW19 effectively replicates only in the respiratory tract and causes mild weight loss in mice.

Pigs, crucial influenza virus hosts, are susceptible to avian and human influenza viruses, making them “mixing vessels” that produce new types of pandemic influenza viruses. Moreover, the SIV subtypes H1N1, H1N2, and H3N2 are prevalent in pig herds and can cause the emergence of various reassortant isolates. Moreover, H3N2 influenza viruses containing the H1N1 internal genes have been widely discovered in Southern China; these viruses, including completely human-origin viruses, early-stage or recent double-reassortant human viruses, and avian-origin viruses, include at least four H3N2 genotypes [41–44]. In the analysis of five H3N2 SIV strains isolated from Guangdong, Southern China, in 2005, the aforementioned virus strains were noted to originate from human-like H3N2 virus strains in the 1990s: H3N2 reassortant virus from humans and H1N1 virus from pigs (both extremely common among pig populations) [45]. Thus, pigs are intermediate hosts for avian-origin viruses before human infection, and they can be “mixing vessels” for genetically reassortant virus generation. We previously reported that the H1N2 virus circulating in pig herds in Zhejiang, Eastern China, is multireassortant with genomes derived from both H1N1 pdm/2009 and swine-origin H3N2 viruses [46]. In general, SIVs circulating in pig herds in Southern and Eastern China have undergone extensive reassorting processes over a long period.

The respiratory tracts of pigs have both avian- and human-like receptors and thus can become infected with either avian or human influenza viruses. Therefore, one of the most common, conventional cross-species transmission routes for avian influenza involves pigs. Moreover, pigs may be naturally infected with avian-origin influenza viruses, including the H4N6 virus, the H9N2 virus, and the highly pathogenic H5 viruses [47–50]. Considering the large scale of poultry and pig farming, various SIV and avian influenza virus subtypes have been widely discovered in China, highlighting that the potential threats of SIVs to human health may continue to exist. In the current study, ZJ-SW19 was noted to carry five pmd/2009 internal segments, suggesting that influenza virus reassortment occurs in pig populations in Eastern China. Multiple antigenic drifts have introduced H3N2 SIVs to humans over a long period [51]. Thus, further continual monitoring and research are warranted.

In summary, we genetically and biologically characterized the novel reassortant H3N2 SIV isolated from pig farms. Our results suggested that this SIV can bind to human- and avian-like receptors along with some mammal-adapted mutations; it can effectively replicate in the lungs in mice, exhibiting moderate virulence. Taken together, our results highlight the necessity for continued surveillance of H3N2 SIVs in Eastern China to prevent any related public health emergencies.

## Data Availability

The datasets used and/or analyzed during the current study are available from the corresponding author upon reasonable request.
